# Study of brainstem auditory evoked potentials in early diagnosis of congenital toxoplasmosis^☆^^☆☆^

**DOI:** 10.1016/j.bjorl.2018.03.012

**Published:** 2018-04-22

**Authors:** Aline Almeida Fontes, Sirley Alves da Silva Carvalho, Gláucia Manzan Queiroz de Andrade, Ericka Viana Carellos, Roberta Castro Romanelli, Luciana Macedo de Resende

**Affiliations:** a Universidade Federal de Minas Gerais (UFMG), Faculdade de Medicina, Programa de Pós-Graduação em Ciências Fonoaudiológicas, Belo Horizonte, MG, Brazil; b Universidade Federal de Minas Gerais (UFMG), Faculdade de Medicina, Departamento de Fonoaudiologia, Belo Horizonte, MG, Brazil; c Universidade Federal de Minas Gerais (UFMG), Faculdade de Medicina, Departamento de Pediatria, Belo Horizonte, MG, Brazil

**Keywords:** Congenital toxoplasmosis, Hearing, Auditory evoked potentials, Electrophysiology, Toxoplasmose congênita, Audição, Potenciais evocados auditivos, Eletrofisiologia

## Abstract

**Introduction:**

Congenital toxoplasmosis is an infectious disease with high prevalence in tropical countries. It is characterized by neurological, ophthalmological and auditory sequelae.

**Objective:**

The aim of this study was to evaluate and describe the brainstem auditory evoked potential in infants aged 1–3 months diagnosed with congenital toxoplasmosis and to compare them with infants of the same age group without the infection.

**Methods:**

This is an observational, analytical and cross-sectional study in which brainstem auditory evoked potential was investigated in infants with congenital toxoplasmosis. The following audiological exams were performed: transient-evoked otoacoustic emissions, clinical and automatic brainstem auditory evoked potential.

**Results:**

100 children participated in the study, but the final sample consisted of 76 children. Of the 37 children with toxoplasmosis included in the study, 28 completed the neurological imaging evaluation, and of these, 3 (10.7%) showed an altered neurological examination. At the brainstem auditory evoked potential assessment, two children without toxoplasmosis and 10 children with congenital toxoplasmosis had results suggestive of alterations in the brainstem auditory pathway maturation.

**Conclusion:**

10 (27%) children were identified with a possible unilateral alteration in the electrophysiological assessment. There was a 5-fold higher risk for a child between 1 and 3 months of age with toxoplasmosis to have an altered brainstem auditory evoked potential compared to a child of the same age range without the infection.

## Resumo

**Introdução:**

A toxoplasmose congênita é uma doença infecciosa com grande prevalência nos países tropicais. Caracteriza-se por sequelas neurológicas, oftalmológicas e auditivas.

**Objetivo:**

O objetivo desse estudo foi avaliar e descrever o potencial evocado auditivo de tronco encefálico em bebês de 1 a 3 meses diagnosticados com toxoplasmose congênita e comparar com bebês de mesma faixa etária sem a infecção.

**Metódo:**

Trata-se de um estudo observacional, analítico e transversal, no qual foi realizada a pesquisa do potencial evocado auditivo de tronco cerebral em lactentes com toxoplasmose congênita. Foram realizados os exames audiológicos: emissões otoacústicas evocadas por estímulo transiente, potencial auditivo de tronco encefálico clínico e automático.

**Resultados:**

Participaram do estudo 100 crianças, porém a amostra final foi constituída por 76. Das 37 crianças com toxoplasmose incluídas no estudo, 28 completaram a avaliação neurológica de imagem e, dessas, três (10,7%) apresentaram exame neurológico alterado. Na avaliação do potencial evocado auditivo de tronco encefálico, duas crianças sem toxoplasmose e 10 com toxoplasmose congênita apresentaram resultado sugestivo de alteração no processo maturacional da via auditiva de tronco encefálico.

**Conclusão:**

Foram identificadas 10 (27%) crianças com possível alteração unilateral na avaliação eletrofisiológica e um risco cinco vezes maior de uma criança entre um e três meses com toxoplasmose apresentar alteração no potencial evocado auditivo de tronco encefálico quando comparada com uma criança da mesma faixa de idade sem a infecção.

## Introduction

Congenital toxoplasmosis (CT) is an infectious disease caused by the transmission of the protozoan *Toxoplasma gondii* (*T. gondii*) through the placenta to the fetus. The disease occurs mainly after a mother's primary infection during pregnancy, and, to a lesser extent, through the reactivation of a previous infection in mothers with immunodeficiency. The disease is included in the TORCHS (Toxoplasmosis, Rubella, Cytomegalovirus, Herpes simplex and Syphilis) group of congenital infections, which represent a risk for the development of auditory alterations.^1^, ^2^, ^3^

The infection has a variable occurrence rate from region to region, with a higher prevalence in tropical countries, and is common in Central and South America. The prevalence rate is associated with warm climates, food and hygiene habits adopted by humans, and the population of cats, which are the definitive host of the protozoan *T. gondii*.^4^ In a study^1^ carried out in São Paulo, Brazil, it was observed that the incidence of congenital toxoplasmosis ranged from 9.5 to 10.6 per 1000 births. In Belo Horizonte (Brazil), the prevalence was one case per 1590 live births.^5^ In the city of Recife (Brazil), the frequency of toxoplasmosis in newborns whose mothers had the acute infection during pregnancy was 12.5%.^6^ This prevalence can vary according to the diagnostic methodology used.

In cases of congenital toxoplasmosis, most children are born asymptomatic or show nonspecific clinical manifestations. The disease morbidity is associated with neurological sequelae (brain calcifications and/or ventricular dilatation) that occur in approximately ⅓ of cases, and ophthalmological sequelae (retinochoroiditis), which are present in about 80% of affected children in Brazil.^7^ The most severe neurological forms are mainly related to the vertical transmission in the first trimester of gestation. Recent studies^8^, ^9^, ^10^, ^11^, ^12^ demonstrate that congenital toxoplasmosis is also a potential risk factor for deafness, and sensorineural hearing loss has been reported in children with this infection. However, the results are variable and there is no consensus regarding hearing impairment related to this parasitic infection.

A study^12^ carried out in 1980 in the United States, which evaluated 24 children with subclinical congenital toxoplasmosis, identified cases of unilateral and bilateral sensorineural hearing loss. In 1996, in the Middle East, another study^13^ observed a higher prevalence of sensorineural hearing impairment in children at risk for *T. gondii* infection.

These findings can be explained by the inflammatory process triggered by the parasite, which can also affect the inner ear, with consequent calcification during the resolution process. Kelemen^14^ found calcifications in the spiral ligament and vascular stria of the cochlea in the right ear of a newborn with congenital toxoplasmosis that also had cortical calcifications. The author stated they were characteristic findings of congenital infection by *T. gondii*.

Most studies that investigated the incidence of hearing problems related to congenital toxoplasmosis mentioned cochlear disorders with auditory acuity impairment.^8^, ^12^, ^13^ A study^5^ involving children diagnosed with congenital toxoplasmosis in the state of Minas Gerais, Brazil, verified that 21.1% of the assessed children had sensorineural hearing loss. Little research has been performed on the occurrence of central hearing impairment in children with congenital toxoplasmosis. As previously mentioned, neurological disorders are common in this population and, therefore, it is logical to investigate central auditory function to allow us to infer the possible negative consequences for the development of communication.

The investigation of possible lesions or dysfunctions in the Central Auditory Nervous System can be achieved by evaluating the Auditory Evoked Potentials (AEPs). AEPs evaluate neuroelectric activity and integrity of the auditory pathways from the auditory nerve to the cerebral cortex in response to an acoustic stimulus and have been used as an objective tool for auditory diagnosis.^15^, ^16^, ^17^ AEPs are classified as short, medium or long-latency. The short-latency AEP, most frequently used and indicated for auditory diagnosis in infants, is the Brainstem Auditory Evoked Potential (BAEP).^18^ This examination is an objective one, does not require attention or response from the patient, is used for the diagnosis and identification of pathologies that affect the auditory pathway integrity to the inferior colliculus in the brainstem.

The studies performed in children with and without risk of hearing loss have used the evoked otoacoustic emissions (OAEs) associated with BAEP as important tools in audiological diagnosis, principally for the identification of the auditory neuropathy spectrum,^17^, ^18^, ^19^, ^20^, ^21^ which is characterized by dysfunction affecting the auditory nerve. The most classic clinical characteristic of auditory neuropathy is a normal cochlear function, with the presence of OAE and altered neural function, with altered or absent BAEP responses from wave I on.^21^

The aim of this study was to evaluate and describe the BAEP results in infants aged 1–3 months old diagnosed with congenital toxoplasmosis and to compare them with the audiological parameters of infants at the same age group but without a diagnosis of the infection.

## Methods

This is an observational, analytical and cross-sectional study, in which BAEP was investigated in infants with congenital toxoplasmosis. The test results were compared in two groups of children aged 1 to 3 months, with and without a diagnosis of congenital toxoplasmosis.

Infants aged 1 to 3 months participating in the Congenital Toxoplasmosis Control Program in Minas Gerais (PCTC-MG) – *Núcleo de Ações e Pesquisa em Apoio Diagnóstico* – (NUPAD/UFMG) were invited to participate in the study from February to December 2015. All assessed infants had positive anti-*T. gondii* IgM antibody test in the neonatal screening test in dried-blood specimens and underwent serology (anti-*T. gondii* IgM, IgA and IgG) to confirm congenital toxoplasmosis. The final diagnosis had not yet been established on the day of the audiological assessment. Infants at the same age group, without a diagnosis of congenital infection, were also invited to participate in the study, who were rooming-in at the maternity unit of a referral public state hospital participating in the Universal Neonatal Hearing Screening Program (TANU) of Hospital das Clínicas of UFMG (HC-UFMG) and participants of the Universal Neonatal Hearing Screening Program (TANU).

For sample size calculation, the prevalence estimation formula for simple random samples was used. The expected prevalence of hearing loss was 21%, as described in a previous study.^5^ The sample calculation indicated the number of 75 children. It was decided to start the study with 100 children, considering the potential for loss during the follow-up.

Congenital toxoplasmosis was confirmed by the presence of IgM and/or IgA in the serum associated with the presence of specific IgG; or by the presence of IgG associated with clinical manifestations of the infection. As part of the protocol for the monitoring of these children by the Toxoplasmosis Control Program, transfontanelle ultrasonography was performed and, when there was clinical indication, computed tomography of the skull was also performed to assess the neurological impairment, as well as indirect retinoscopy (examination of the eye fundus) to assess ocular impairment. The children with a confirmed diagnosis were treated with sulfadiazine, pyrimethamine and folinic acid during the first year of life, according to an international consensus.^22^ Children with a highly probable diagnosis were treated with the same regimen and had the serology repeated until diagnosis confirmation or exclusion.

At the end of the recruitment, two groups were created with infants aged 1–3 months of both genders: Group 1 with a presumed diagnosis of congenital toxoplasmosis and Group 2 without congenital toxoplasmosis.

All the mothers of the participating infants had a minimum of six prenatal consultations during pregnancy, no health complications, and did not use any medications during pregnancy, according to information collected at the interviews prior to the creation of the study groups.

For Group 1, the inclusion criteria were: accepting and signing the Free and Informed Consent form (FICF), confirmed diagnosis of Congenital Toxoplasmosis, age between 1 and 3 months and presence of Transient Evoked Otoacoustic Emissions (TEOAE) in at least one ear. For Group 2: accepting and signing the FICF, ruled out or unlikely diagnosis of congenital toxoplasmosis, 1–3 months of age, and absence of additional risk indicators for hearing loss.^3^

The ears with absent TEOAE were excluded from the final study analysis, to rule out possible conductive alterations, as well as ears in which it was not possible to perform and/or complete the audiological exams.

The auditory evaluations were performed at the Speech and Audiology Outpatient Clinic of the Hospital das Clínicas of UFMG, in an acoustically isolated room, in the presence of parents and/or caregivers. They were performed with the infants in natural sleep, with artifact level control to obtain reliable records.

All children underwent auditory evaluation that included the TEOAE, BAEP and automatic BAEP procedures. The ears that showed the presence of TEOAE were included for BAEP analysis. Some BAEP exams were excluded from the analysis due to the presence of muscle artifact, impedance difficulty, equipment probe intolerance that made it difficult to analyze and/or complete the exam. Children who showed no response to TEOAE, BAEP and/or failed to complete the assessment were retested and those who maintained the response alteration in the exams were referred for audiological diagnosis.

To perform the audiological tests, the Elios^®^ equipment manufactured by Echodia (Clermont-Ferrand, France) was used. Before the BAEP, the TEOAE was investigated to evaluate peripheral cochlear function. The TEOAE assessment was performed with non-linear click stimulus and 80 dB SPL intensity in a 20 ms window. An artifact rejection rate of 20 mPa was established to record emissions.

The BAEP was performed with alternating click stimuli with a duration of 0.1 ms, presented at a rate of 17.1 stimuli per second at an intensity of 80 dB HL. A one-channel recording was performed, with a 50,000 gain in a 15 ms analysis window, high pass filter of 30 Hz and low pass of 1500 Hz and positioning the electrodes in Fz as the active electrode, M1 and M2 as negative electrodes and Fpz as the ground electrode.

Two 1000-stimulus scans were performed to investigate the integrity of the auditory pathway at the intensity of 80 dB. A specific neonatal screening protocol was also performed, with a 40-dB pass/fail, to verify the presence or absence of V wave at this intensity. The analysis of the BAEP latency measures was performed by blinded study investigators.

The study and the FICF were approved by the Research Ethics Committee of UFMG under Opinion 810,127. This study is part of a project carried out by the Brazilian Congenital Toxoplasmosis Group of UFMG (with the support of NUPAD-UFMG), which is responsible for the State Neonatal Screening Program in the state of Minas Gerais.

The continuous variables assessed were: absolute latency of waves I, III and V, interpeak intervals I-III, III-V, I-V and V-wave interaural latency at 80 dB. The categorical variables were: presence of congenital *T. gondii* infection, presence or absence of neurological sequelae (ventricular calcifications and/or dilatation), presence or absence of wave V at 40 dB, child's age (1 month, 2 months or 3 months) and normal or potentially abnormal BAEP. A response with possible alteration was considered when one of these conditions was observed: (1) absence of wave V at 40 dB HL; (2) increase in latency or interpeak intervals, considering as reference the mean +1.5 standard deviation of Group 2.

For the statistical analysis, the SPSS program, version 15.0 (SPSS, Inc., Chicago, IL, United States of America) was used. A descriptive analysis of the exam results was carried out and an association was verified between the results obtained in the different study groups. The Shapiro–Wilks normality test was performed for the continuous variables, and, in the presence of a non-normal distribution, the Kruskal–Wallis test was used to compare groups of continuous variables. For the dichotomous variables, the chi-square test or Fisher's exact test was used. A level of significance of 5% was considered for the statistical analysis.

## Results

### Characteristics of the study participants

**Figure 1:**
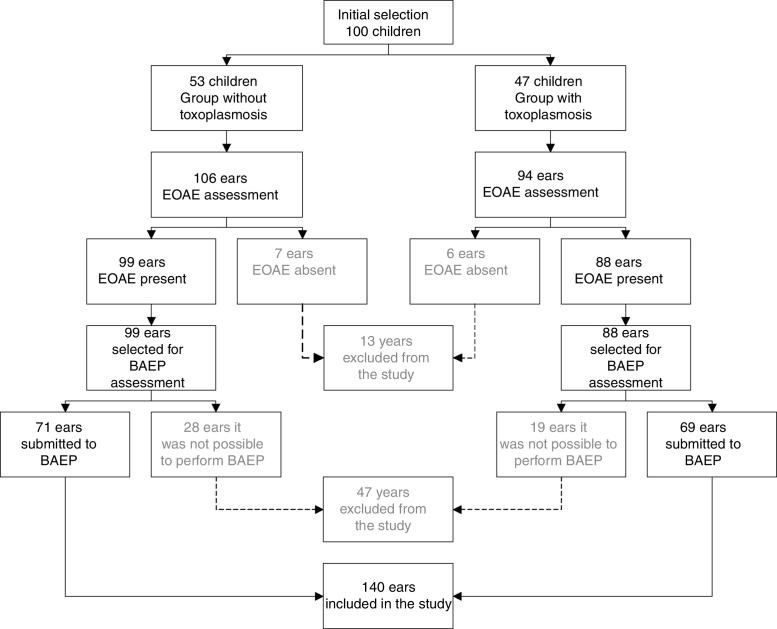
Flowchart of the distribution of the children participating in the study according to the presence or absence of congenital toxoplasmosis, and number of respective ears included and excluded. EOAE, evoked otoacoustic emissions; BAEP, brainstem auditory evoked potential.

From February to December 2015, the parents of the infants participating in the Congenital Toxoplasmosis Control Program in the state of Minas Gerais and treated at the UFMG Hospital das Clínicas, were invited to participate in this study if their infant had a positive or equivocal neonatal screening test for toxoplasmosis; also invited were parents of the babies born in the maternity unit of HC-UFMG, according to the order of arrival. A total of 133 parents of infants were invited, with 33 infants being excluded from the study due to non-attendance on the days and times scheduled for the audiological evaluation. To determine whether the patient was at risk for hearing loss, data were collected on family, gestational and infant health history. In the end, 100 children were included in the study. The number of ears of these children that were included or excluded is described in Fig. 1.

For the final analysis, the sample consisted of 76 children between 1 month and 3 months of age, 37 (48.7%) with congenital toxoplasmosis and 39 (51.3%) without congenital infection (controls). Of the 37 babies with toxoplasmosis, 21 (57%) were females and 16 (43%) were males. Of the 39 infants without a diagnosis of infection, 20 (51%) were females and 19 (49%) were males. The mean age and standard deviation (SD) were 1.87 months (SD = 0.92) for the control group and 1.81 months (SD = 0.70) for the group with congenital toxoplasmosis. The groups were similar regarding age (*p* = 0.747) and gender (*p* = 0.653). Of the 37 children with CT in the study, 28 underwent imaging exams for neurological evaluation before the date of analysis, and of these, 3 (10.7%) had altered neurological examination. The neurological alterations found in these children by transfontanelle ultrasonography and/or computed tomography were: diffuse multiple brain calcifications, ventricular dilatation, and increased protein levels in the cerebrospinal fluid (hyperproteinorrachia). Of these three children, one had an altered BAEP result response.

### Responses to brainstem auditory evoked potential

As shown in the flowchart (Fig. 1), 47 ears were excluded from the study due to the inability to record the BAEP, due to an artifact level higher than 12%, difficulty in obtaining impedance that compromised the analysis of wave morphology and/or incomplete examination.

The comparative analysis of the wave I, III and V latency responses of the right and left ears showed no statistically significant differences (*p* > 0.05), which allowed an independent analysis of the ear. A total of 140 ears was included the study, 69 from the toxoplasmosis group and 71 from the control group. The analysis indicated a lack of statistical difference when comparing the absolute and interpeak latency between the groups with and without a diagnosis of congenital toxoplasmosis (Table 1).

**Table 1:** Comparative analysis of the BAEP results in children with and without congenital toxoplasmosis (*n* = 140).

Overall	Wave I absolute latency	Wave III absolute latency	Wave V absolute latency	I–III interpeak interval	III–V interpeak interval	I–V interpeak interval
*Without toxoplasmosis (n* *=* *71)*						
Mean	1.65	4.24	6.41	2.59	2.17	4.76
SD	0.20	0.37	0.40	0.39	0.39	0.42
Minimum	1.28	3.41	5.50	1.63	1.22	3.94
Maximum	2.09	4.97	6.97	3.45	2.97	5.59
Median	1.66	4.16	6.44	2.62	2.18	4.75

*With toxoplasmosis (n* *=* *69)*						
Mean	1.61	4.21	6.45	2.61	2.23	4.85
SD	0.15	0.33	0.37	0.31	0.35	0.34
Minimum	1.16	3.31	5.56	1.91	1.38	3.87
Maximum	1.94	4.91	7.03	3.40	3.25	5.56
Median	1.65	4.19	6.40	2.62	2.22	4.88
*p* value^a^	0.22	0.91	0.63	0.64	0.41	0.17

*n*, number of ears; SD, standard deviation; *p*, significance probability.

a Kruskal–Walls test.

Considering that the child's age is a determinant for the BAEP wave latency value, Table 2 indicates the comparison of the latency and interpeak values of the children with and without a diagnosis of toxoplasmosis considering the ages of 1 month, 2 months and 3 months.

**Table 2:** Comparison of absolute latencies and interpeak intervals of the ears of children with and without congenital toxoplasmosis considering the ages of one month, two months and three months (*n* = 140 ears).

Absolute latency and interpeak	1 month			2 months			3 months		
	Control group *n* = 34	CT group *n* = 24	*p* value^a^	Control group *n* *n* = 11	CT group *n* = 35	*p* value^a^	Control group *n* *n* = 26	CT group *n* = 10	*p* value^a^
	Mean (SD)	Mean (SD)		Mean (SD)	Mean (SD)		Mean (SD)	Mean (SD)	
I	1.67 (0.19)	1.64 (0.37)	0.327	1.62 (0.22)	1.59 (0.16)	0.606	1.61 (0.22)	1.60 (0.12)	0.804
III	4.34 (0.33)	4.15 (0.33)	0.647	4.26 (0.30)	4.19 (0.31)	0.544	4.08 (0.39)	4.1 (0.40)	0.874
V	6.48 (0.34)	6.65 (0.30)	0.039^a^	6.39 (0.38)	6.35 (0.31)	0.718	6.32 (0.41)	6.28 (0.43)	0.903
I–III	2.66 (0.35)	2.61 (0.30)	0.71	2.64 (0.30)	2.63 (0.29)	0.939	2.46 (0.43)	2.54 (0.38)	0.768
III–V	2.13 (0.39)	2.39 (0.30)	0.06^a^	2.12 (0.36)	2.14 (0.31)	0.99	2.14 (0.44)	2.13 (0.48)	0.543
I–V	4.80 (0.37)	5.01 (0.26)	0.014^a^	4.77 (0.33)	4.78 (0.32)	0.82	4.70 (0.50)	4.68 (0.42)	0.768

SD, standard deviation; *p*, significance probability; CT, congenital toxoplasmosis; *n*, number of ears in each group by age group.

a Kruskal–Walls test.

The results indicate that the mean wave V absolute latency value decreases as the child's age increases. The comparison between the mean value of the BAEP wave V of the group with toxoplasmosis and without toxoplasmosis showed a difference with statistical relevance when comparing the children with one month of age, indicating that the mean values of the V wave for one-month aged children with toxoplasmosis was higher than the values of the same wave for children without toxoplasmosis (Table 2). At the same age range, there was also a statistical difference when comparing the I-V interpeak latency, and close to the level of significance for the III-V interpeak interval between children with and without toxoplasmosis (Table 2).

Aiming to classify the results of the BAEP as normal or with a possible alteration, the mean value plus 1.5 of the standard deviation of the control group values was considered as the cutoff point. The cutoff points used in the study are shown in Table 3.

**Table 3:** Reference values for the classification of BAEP responses as normal or with possible alteration at one month, two months and three months.

Age	Characteristics	Absolute latency			Interpeak interval		
		Wave I	Wave III	Wave V	I–III	III–V	I–V
1 month	Mean of Control Group	1.67	4.34	6.48	2.66	2.13	4.80
	Standard Deviation	0.19	0.33	0.34	0.35	0.39	0.37
	Mean + 1.5 SD	1.96	4.84	6.99	3.19	2.72	5.36
2 months	Mean of Control Group	1.62	4.26	6.39	2.64	2.12	4.77
	Standard Deviation	0.22	0.30	0.38	0.30	0.36	0.33
	Mean + 1.5 SD	1.95	4.71	6.96	3.09	2.66	5.27
3 months	Mean of Control Group	1.61	4.08	6.32	2.46	2.24	4.70
	Standard Deviation	0.22	0.39	0.41	0.43	0.44	0.50
	Mean + 1.5 SD	1.94	4.67	6.94	3.11	2.90	5.45

SD, standard deviation.

Based on the values indicated in Table 3, each ear was classified as normal or with a possible alteration when it showed an increase in absolute or interpeak latency of any of the analyzed waves. Additionally, ears with absent wave V formation at 40 dB were also considered as having a possible alteration. Table 4 indicates the comparison of the proportion of altered ears between the groups with and without toxoplasmosis.

**Table 4:** Comparison of the proportion of ears with normal and altered BAEP between the groups with toxoplasmosis and without toxoplasmosis (*n* = 140).

BAEP results	Without toxoplasmosis *n* (%)	With toxoplasmosis *n* (%)	*p*^a^	RR (CI)
Normal	69 (97)	59 (86)	0.014	5.84 (1.2–27)
Altered	2 (3)	10 (14)		

*n*, number of participants; *p*, significance probability; RR, relative risk; CI, confidence interval.

a Chi-squared test.

Two altered ears were identified in the group without toxoplasmosis and 10 altered ears in the toxoplasmosis group. Comparison of the proportion of alterations between the groups indicated a statistical difference (*p* = 0.014). It was observed that a child with toxoplasmosis is 5-fold more likely to have an altered BAEP compared to a child without toxoplasmosis.

The alterations found in the BAEP of the group without a diagnosis of toxoplasmosis were: (1) Ear with increased wave III latency and increased I-III interpeak interval (*n* = 1); (2) Increased I-V interpeak interval (*n* = 1). In the group with toxoplasmosis, the alterations found in the BAEP were: (1) Ear with absence of wave V formation at 40 dB (*n* = 1); (2) Ear with increased wave I latency (*n* = 1); (3) Ears with increased wave III latency and I-III interpeak interval (*n* = 4); (4) Ears with increased I-III interpeak interval (*n* = 2); (5) Ear with increased III-V interpeak interval (*n* = 2).

The comparison between wave V interaural difference in relation to the groups indicated no statistical difference (Table 5).

**Table 5:** Comparison of the wave V latency difference between the right and left ears in relation to the groups.

Interaural difference (Wave V)	Without toxoplasmosis	With toxoplasmosis	*p*^a^
Mean	0.22	0.16	*p* = 0.064
SD	0.15	0.15	
Minimum	0.00	0.00	
Maximum	0.68	0.56	
Median	0.19	0.12	

a Kruskal–Walls test.

Of the 37 children with congenital toxoplasmosis assessed by BAEP, 10 (27%) children showed BAEP results suggestive of abnormality, 1 child with possible unilateral auditory impairment, characterized by absence of wave V at 40 dB at BAEP and 9 children with possible unilateral impairment, characterized by increased latency and/or interpeak interval at BAEP, according to the reference criterion.

Of the 10 children with possible auditory impairment, one had altered neurological examination (diffuse brain calcifications and hyperproteinorrachia), three did not have the definitive neurological diagnosis at the time of the analysis and six had a normal neurological imaging examination.

## Discussion

It is a consensus in the literature that congenital toxoplasmosis has retinochoroiditis and neurological lesions (brain calcifications) as the most frequent clinical manifestations.^1^, ^4^, ^6^, ^8^

As with other TORCHS infections, toxoplasmosis is also associated with the risk of developing hearing loss.^23^, ^24^, ^25^, ^26^, ^27^

Studies evaluating newborns with congenital toxoplasmosis showed that most children are asymptomatic, with rates ranging from 65%^5^ to 71.3%.^28^ However, when more detailed investigations are performed, neurological alterations such as intracranial calcifications and ophthalmological lesions can be found,^26^ as well as auditory alterations.

Children with congenital toxoplasmosis may also have severe neurological sequelae, with rates ranging from 17.9% to 64%.^12^, ^29^, ^30^ In the present study, 10.7% of the babies with toxoplasmosis had neurological sequelae (diffuse brain calcifications and hyperproteinorrachia), and 27% had BAEP results suggestive of some unilateral auditory alteration.

Studies show that early diagnosis associated with drug treatment may benefit the child's development and improve prognosis. The treatment should be initiated as early as the first days of life of the newborn with aa combination of sulfadiazine, pyrimethamine and folinic acid, and should be maintained during the first 12 months of the child's life.^2^, ^29^ In the present study, all children with confirmed or very probable congenital toxoplasmosis began the drug treatment and had their hearing evaluated until 3 months of age.

The Universal Neonatal Hearing Screening Program (TANU) is performed with the objective of identifying newborns with a higher probability of hearing loss.^31^ As previously mentioned, children with congenital toxoplasmosis are at risk for hearing loss and may present with late auditory deficit.^5^, ^23^, ^26^

The TANU has been carried out through the use of TEOAE and BAEP. Although it is an examination that requires more time for its application, the use of BAEP is necessary to rule out false-positive results, as in the case of babies with present TEOAE and are considered as having normal hearing; however, when their hearing is investigated through BAEP, they may be diagnosed as having retrocochlear disorders, such as the auditory neuropathy spectrum. The auditory neuropathy spectrum is suspected when TEOAE presence is observed, together with the absence of BAEP responses.^16^, ^21^ Therefore, the safest conduct for the early detection of hearing loss is the combined use of TEOAE and BAEP, especially for the at-risk population.^17^, ^20^, ^32^, ^33^

A study^20^ indicated that only 42% of the children screened in the neonatal period with absence of TEOAE and presence of a response in automatic BAEP were diagnosed later with hearing loss. These results confirm that the retest and follow-up of hearing development are important to rule out the false-positive results. Additionally, the combination of the two exams increases the sensitivity and specificity of neonatal auditory screening in the early detection of hearing disorders.

Another study^18^ verified that automatic BAEP has a sensitivity of 100% (all ears diagnosed with hearing loss did not pass the automatic BAEP exam) and specificity of 99.7% (ears with normal hearing passed the automatic BAEP exam). Similar to the mentioned studies, the present study evaluated all infants through TEOAE and BAEP, since they are routinely used in the TANU programs. Babies evaluated in this study who failed any of the exams were referred for retest and auditory diagnosis. Retesting and referral for BAEP are essential in hearing screening to confirm or not the failure and referral for diagnostic evaluation.^33^

The results found here for waves I, III and V and interpeak intervals in the group of children without toxoplasmosis are close to those reported in the literature for children without risk of hearing loss and of the same age group.^34^, ^35^

This study showed that the BAEP result was not different between the right and left ears. This fact had previously been demonstrated in another study,^34^ which found no statistically significant difference in the analysis of the absolute latency of the waves and interpeak intervals between the right and left ears, indicating that the auditory pathway maturation process occurs in a similar way on both sides of the auditory pathway.

In the present study, a comparison of the results of the BAEP analysis, regardless of the ear, was made between the groups of children with and without congenital toxoplasmosis. The comparison showed a statistically significant difference between the groups of children aged one month regarding wave V latency and III-V and I-V interpeak intervals, indicating latency delay in the group with toxoplasmosis in relation to the group without toxoplasmosis.

This result demonstrates a possible association between increased BAEP latency and *T. gondii* infection, further highlighting the risk of these children developing a possible auditory impairment due to delay in Central Nervous System maturation.

Other studies^12^, ^30^, ^36^ involving children diagnosed with congenital toxoplasmosis have shown some type of hearing alteration, such as sensorineural hearing loss and altered auditory processing. In the present study, BAEP results suggestive of hearing alteration were found in 10 children with congenital toxoplasmosis. Aiming to classify the child as having normal or altered hearing, the absence of the wave V at 40 dB was considered, and the cutoff point was also used, having as reference the mean value added to 1.5 of standard deviation of the control group according to each age range. Studies have shown that age is a determinant in the latency value and the interpeak interval of BAEP, due to the brainstem auditory pathway maturational process that occurs until the second year of life.

Based on this classification, two children with altered results (2 ears) were identified in the control group and 10 altered children (10 ears) were identified in the toxoplasmosis group. These results indicated an increased risk for a child with congenital toxoplasmosis to develop auditory alterations compared to children without a diagnosis of the infection. The alterations found were: ear with absence of wave V formation at 40 dB (suggestive of impaired hearing acuity or peripheral cochlear disease); ear with increased wave I latency (suggestive of auditory neuropathy); ears with increased wave III latency (suggestive of maturational delay) and increased I-III interpeak interval (suggestive of maturational delay); ears with increased I-III and III-V interpeak intervals (suggestive of central dysfunction).

These responses should not be interpreted as proof of auditory impairment, since they may have been a consequence of a dysfunction in the infant's auditory pathway development, which is still undergoing the maturational process, but they are signs of a possible myelination alteration or central/retrocochlear dysfunction. These findings indicate the need for regular monitoring of the hearing abilities of children with toxoplasmosis. Monitoring the hearing and language development of these children may, in the future, demonstrate the prognostic value and the importance of early identification of BAEP alterations.

Some limitations were evident when carrying out this study, such as: the number of losses (exclusions) due to the non-successful performance of the TEOAE and the BAEP and the non-inclusion of the final auditory diagnosis performed in infants who had shown altered results in one of the performed exams. These limitations should be considered and minimized in other studies, but they do not decrease the importance of this study in evaluating young infants with congenital toxoplasmosis from all over the state, in a region where toxoplasms with higher pathogenicity predominate.^37^

The present study showed the importance of investigating hearing at an early age and monitoring auditory development in children with congenital toxoplasmosis, aiming to eliminate future auditory alterations that can develop later in life, thus promoting a better quality of life in this population.

## Conclusion

It was verified that 27% (*n* = 10) of the children aged 1 to 3 months with toxoplasmosis were identified as having a possible unilateral alteration in the BAEP and are 5-fold more likely to have an alteration in the BAEP than children at the same age range without the infection.

## Funding

Study carried out at the Postgraduate Program in Speech and Hearing Sciences, Faculdade de Medicina, Universidade Federal de Minas Gerais – UFMG, Belo Horizonte (MG), Brazil; with a grant provided by Coordenação de Aperfeiçoamento de Pessoal de Nível Superior (CAPES).

## Conflicts of interest

The authors declare no conflicts of interest.
